# The impact of disease-modifying therapies on immunoglobulin blood levels in patients with multiple sclerosis: a retrospective cross-sectional study

**DOI:** 10.1177/17562864231162661

**Published:** 2023-04-17

**Authors:** Ana Klein, Martina Flaskamp, Achim Berthele, Friederike Held, Harisa Muratovic, Bernhard Hemmer

**Affiliations:** Department of Neurology, School of Medicine, Technical University of Munich, Klinikum rechts der Isar, München, Germany; Department of Neurology, School of Medicine, Technical University of Munich, Klinikum rechts der Isar, München, Germany; Department of Neurology, School of Medicine, Technical University of Munich, Klinikum rechts der Isar, München, Germany; Department of Neurology, School of Medicine, Technical University of Munich, Klinikum rechts der Isar, München, Germany; Department of Neurology, School of Medicine, Technical University of Munich, Klinikum rechts der Isar, München, Germany; Department of Neurology, School of Medicine, Technical University of Munich, Klinikum rechts der Isar, Ismaninger Str. 22, München, 81675, Germany; Munich Cluster for Systems Neurology (SyNergy), Munich, Germany

**Keywords:** disease-modifying therapy, hypogammaglobulinemia, immunoglobulins, multiple sclerosis

## Abstract

**Background:**

Although disease-modifying therapies (DMTs) in multiple sclerosis (MS) are known to target the immune system, mechanisms of action, efficacy, safety, and tolerability profiles differ. The long-term impact of DMTs on the immune system and its relation to infectious complications is still poorly understood.

**Objectives:**

To analyze the effect of DMTs on serum immunoglobulin (Ig) levels under consideration of patient demographics and therapy duration.

**Design:**

We included 483 patients on DMTs, 69 patients without DMTs, and 51 controls in this retrospective cross-sectional study.

**Methods:**

IgG, IgM, and IgG subclass 1–4 levels of patients with MS under treatment with DMTs were compared with treatment naive MS patients and controls by multivariate linear regression. Further, Ig levels stratified by DMTs were analyzed regarding therapy duration.

**Results:**

MS patients treated with fingolimod (FG), natalizumab, and B-cell depleting therapies (BCDT) demonstrated significantly lower IgG and IgM levels than healthy controls after a median treatment of 37, 31, and 23 months, respectively (p < 0.05). Treatment with dimethyl fumarate (DMF) and teriflunomide was associated with lower IgG, but not IgM levels. DMF and BCDT were also associated with lower IgG1 levels, while FG led to a reduction of IgG2. Treatment with interferon-beta (IFN) and glatiramer acetate (GA) had no impact on Ig levels. Analysis of subgroups by linear regression also showed a time-dependent decrease of Igs levels in patients treated with BCDT with a median annual reduction of IgG of 3.2% and IgM of 6.2%.

**Conclusion:**

Treatment with DMTs, except GA and IFN, was associated with a decrease in Ig levels. DMTs differed in the extent of decreasing Ig levels but also in their differential effects on Ig subclasses. Monitoring of Ig levels should be considered in patients on long-term treatment with DMTs, particularly those on BCDT, to identify patients at risk of low immunoglobulin levels.

## Introduction

During the past few decades, several disease-modifying therapies (DMTs) for multiple sclerosis (MS) have been approved worldwide. Although these therapies are all known to target the immune response in MS, mechanisms of action, and efficacy, safety and tolerability profiles differ.

Many of these DMTs lead to an increase of infectious complications. However, little is known on the effect of DMTs on immunoglobulin (Ig) serum levels. Of all DMTs administered in MS most is known in regard to rituximab and its effect on immunoglobulin serum levels. Rituximab, a B-cell depleting therapy (BCDT), is used for treating many autoimmune-mediated and hematological diseases. Various studies have shown an association between low levels of both IgG and IgM and rituximab therapy.^1–7^ Two recent studies evaluating the long-term effect of rituximab on Ig levels in patients with neuromyelitis optica spectrum disorders discovered reductions in both IgG and IgM over time.^8,9^ In some studies, a prolonged hypogammaglobulinemia even years after the last treatment with rituximab was demonstrated.^5,10^

In 2017, ocrelizumab, a slightly modified BCDT, was approved for use in patients with MS. Data on its impact on Ig levels are still sparse. However, an initial report on the pivotal Phase III trials of ocrelizumab and their open-label extensions showed a reduction of IgG of approximately 17%, of IgM of between 56% and 58%, and IgA of 20% and 21% over the course of 5.5 years under therapy with ocrelizumab.^11^ Furthermore, a comparative study on treatment outcomes over the first year with rituximab and ocrelizumab showed a continuous reduction of IgG levels in patients under treatment with ocrelizumab, while they remained stable under rituximab. IgM levels were shown to drop similarly under both drugs.^12^

Regarding further DMTs used in the treatment of MS, one recent study showed reduced serum levels of IgG and IgM in patients with MS in comparison to healthy controls. Within this cohort, rituximab, natalizumab (NZ), and fingolimod (FG) were associated with significantly reduced immunoglobulin levels. A multivariable linear regression, however, showed no time dependency of hypoimmunoglobulinemia.^13^ Similar results were found in a further study in regard to NZ, displaying significantly decreased levels of both immunoglobulins in serum. This study furthermore demonstrated a significant time dependency.^14^

So far, only sparse data have been published in regard to the effect of DMTs on the individual IgG subclasses 1–4 and most of these data refer to rituximab. Several studies showed that rituximab leads to a reduction of IgG4 explaining the effective use in combatting IgG4-related systemic diseases.^15–17^

The aim of this study was to identify the effect of the most commonly used DMTs, namely interferon-beta (IFN), glatiramer acetate (GA), dimethyl fumarate (DMF), teriflunomide (TFM), FG, NZ, and BCDT (ocrelizumab and rituximab) on IgM, IgG and its subclasses 1–4 in serum of patients with MS in comparison to healthy controls and untreated MS patients.

## Methods

### Study design and inclusion criteria

In this retrospective cross-sectional study, patient data collected at the Department of Neurology of the Technical University of Munich were analyzed regarding their serum Ig levels. We compared IgG, IgM, and IgG subclass 1–4 serum levels between patients with MS with or without immunotherapy (DMT+ *versus* DMT– patients) with normal controls under consideration of patient demographics and therapy duration. Patients treated with rituximab and ocrelizumab were pooled (BCDT) for most analyses because their mode of action and treatment effects are highly similar. Ocrelizumab was only approved in 2017, median therapy duration was 17 months for patients treated with ocrelizumab in comparison to 30.5 in patients treated with rituximab. As our results show that therapy duration plays a vital role on the reduction of Ig levels, a combined analysis of the two reduces the risk of introducing bias.

Inclusion criteria for the MS groups were defined as follows:

1. No corticosteroid therapy or plasma exchange performed within 4 weeks prior to blood sampling.
2. Therapy duration of at least 6 months in DMT+ patients.
3. In patients receiving BCDT maximum time between last infusion and blood sampling was 12 months.
4. In all other DMT+ patients, maximum time between last application of therapy and blood sampling was 1 month.

Normal controls included patients suffering from unrelated neurological disorders such as unspecified headaches or pseudotumor cerebri and not under treatment with any immunomodulatory or immunosuppressive drugs.

Further information on patient demographics can be found in Table 1.

**Table 1.** Patient demographics.

	Patients	Female gender	Age	Therapy duration	Sub-analysis performed
	*n*	*n* (%)	Median [y (range)]	Median [m (range)]	*n* (%)
Total	603	377 (62.5)	40 (18–76)	28 (6–197)	286 (47.4)
Controls	51	35 (68.8)	36 (19–64)	NA	43 (84.3)
DMT–	69	47 (68.1)	34 (18–69)	NA	26 (37.7)
DMT+	483	295 (61.0)	41 (19–76)	28 (6–197)	217 (44.9)
IFN	59	33 (55.9)	42 (19–67)	61 (6–192)	38 (64.4)
GA	59	36 (61.0)	40 (21–62)	33 (6–197)	28 (47.5)
DMF	68	40 (58.8)	40 (22–55)	23 (6–90)	33 (48.5)
TFM	24	10 (41.7)	47 (20–66)	20 (6–78)	8 (33.3)
FG	105	60 (57.1)	39 (21–65)	37 (6–137)	53 (50.5)
NZ	46	37 (80.4)	36 (22–60)	31 (6–165)	31 (66.0)
BCDT	122*	79 (64.8)	45 (19–76)	23 (6–99)	26 (21.3)**

BCDT, B-cell depleting therapy; DMT, disease-modifying therapy; DMT-, therapy naive patients; DMT+, all patients under treatment with DMT; DMF, dimethyl fumarate; FG, fingolimod; GA, glatiramer acetate; IFN, interferon-beta; NZ, natalizumab; TFM, teriflunomide; NA, not applicable; m, months; y, years.

* 74 patients treated with ocrelizumab, and 48 patients treated with rituximab.

** 14 patients treated with ocrelizumab, and 12 patients treated with rituximab.

### Serum analysis

Serum was collected during diagnostic workup between 2010 and 2021. Most of the samples underwent instant diagnostic testing for IgG and IgM by turbidimetry (cobas 8000, Roche, Basel, Switzerland) and additional blood samples were stored for later analysis at −80°C in the biobank of the Department of Neurology. Frozen samples of MS patients or controls with missing data on Ig concentrations were thawed and analyzed with the same method and machine.

For a subset of patients, frozen sera were available in our biobank (inclusion criteria maximum time between measurement of IgG and IgM and blood sampling for further analysis of IgG subclasses 6 months) to test for IgG subclasses 1–4 using a commercially available ELISA kit (IgG Subclass Human ELISA kit, Thermo Fisher Scientific, Waltham, MA, USA), according to the manufacturer’s instructions. Each sample was tested in double wells.

### Statistical analysis

All analyses were performed using RStudio 1.3.1093 (The R Foundation for Statistical Computing, Vienna, Austria). Distribution of quantitative date was described by median and range. Comparison of Ig and Ig subclass levels between groups was performed by multivariate linear regression, including age, gender, and therapy as independent variables. Ig values underwent logarithmic transformation to conform to normality.

To identify a possible time dependency of this Ig-decrease, a multivariate linear regression was performed for each subgroup individually including age, gender, therapy duration and Ig. Results were described using estimate and *p*-values. Significance levels were set at 5%.

To increase statistical power, we decided to collectively analyze data from patients treated with rituximab and ocrelizumab and define these as patients under BCDT.

## Results

### Study cohort

Baseline patient demographics and therapy duration stratified by drug can be found in Table 1.

Of the 603 patients included in this study, 552 suffered from MS, while 51 served as normal controls. Within the group of MS patients, 69 patients did not receive immunotherapy and therefore constituted the DMT– group, while the other 483 patients were defined as DMT+ (the exact distribution of patients among groups can be found in Table 1).

The median age of our cohort was 40 years (range: 18–76 years). Comparison of subgroups by age resulted in the highest median age in patients treated with BCDT (median: 45 years; range: 19–76 years) and lowest median age in DMT-naive patients (median: 34 years; range: 18–69 years). 62.5% (377) of our participants were of female gender. The largest proportion of females was found in the group of patients treated with NZ (80.4%), the lowest in the group of patients treated with TFM (41.7%).

Median therapy duration was 28 months (range: 6–197 months), with the longest therapy duration in the group of patients treated with IFN (median: 61 months; range: 6–192 months).

Sera for sub-analysis of IgG subgroups was available for 286 (47.4%) patients (the exact distribution of patients among groups can be found in Table 1).

### Comparison of IgG and IgM levels between groups

**Figure 1.**
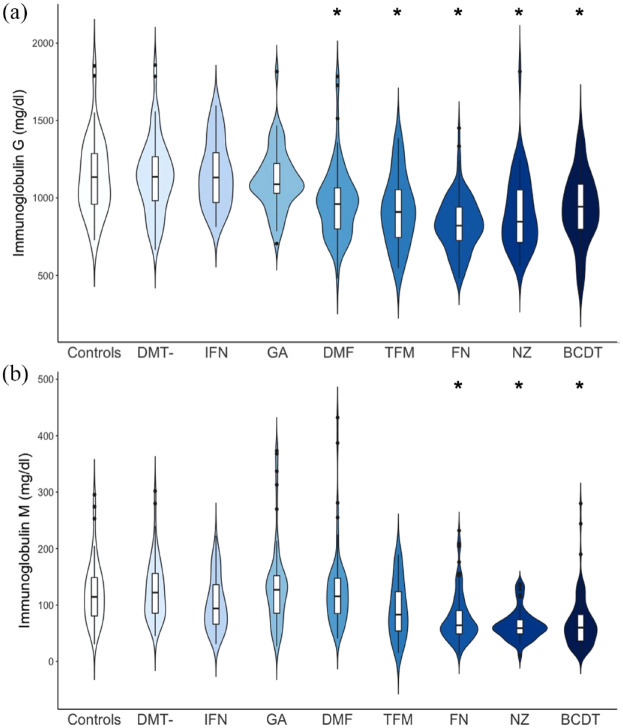
IgG (a) and IgM (b) levels in mg/dl are presented by therapy group in a violin plot, allowing for presentation of data distribution. *Statistical significance compared to controls (see Table 2) BCDT, B-cell depleting therapy, DMF, dimethyl fumarate; DMT, therapy naive patients; FG, fingolimod; GA, glatiramer acetate; IFN, interferon-beta; NZ, natalizumab; TFM, teriflunomide.

Comparison of median Ig levels yielded the highest median IgG concentration in DMT-naive patients (median IgG: 1136 mg/dl; range: 663–1858 mg/dl) and the highest median IgM concentration in patients treated with GA (median IgM: 127 mg/dl; range: 27–373 mg/dl) respectively, while the lowest median IgG level was found in patients treated with FG (median IgG: 821 mg/dl; range: 480–1451 mg/dl) and the lowest median IgM level in patients treated with NZ (median IgM: 59 mg/dl; range: 11–137 mg/dl) respectively (Table 2, Figure 1).

**Table 2.** Median Ig levels by therapy group and results of the multivariate linear regression.

		Median [mg/dl (range)]	Below lower limit of normal* [*n* (%)]	Estimate	*p*-value
Total	IgG	986 (392–1858)	58 (9.6)	NA	NA
	IgM	83 (11–432)	64 (10.6)	NA	NA
Controls	IgG	1134 (727–1853)	0 (0.0)	Reference	Reference
	IgM	114 (30–296)	0 (0.0)	Reference	Reference
DMT–	IgG	1136 (663–1858)	1 (1.4)	−0.001	0.968
	IgM	122 (45–302)	0 (0.0)	0.024	0.563
IFN	IgG	1132 (812–1598)	0 (0.0)	0.018	0.326
	IgM	94 (31–223)	4 (6.8)	−0.029	0.178
GA	IgG	1088 (705–1816)	0 (0.0)	−0.002	0.907
	IgM	127 (27–373)	0 (0.0)	0.029	0.494
DMF	IgG	956 (480–1785)	6 (8.8)	−0.067	**<0.001**
	IgM	116 (41–432)	0 (0.0)	0.019	0.633
TFM	IgG	909 (546–1387)	3 (12.5)	−0.082	**0.001**
	IgM	83 (15–189)	0 (0.0)	−0.104	0.061
FG	IgG	821 (480–1451)	23 (21.9)	−0.132	**<0.001**
	IgM	64 (11–232)	16 (15.2)	−0.204	**<0.001**
NZ	IgG	846 (561–1816)	9 (19.6)	−0.104	**<0.001**
	IgM	59 (11–137)	7 (15.2)	−0.281	**<0.001**
BCDT	IgG	986 (392–1858)	16 (13.1)	−0.081	**0.018**
	IgM	60 (14–280)	32 (26.2)	−0.275	**<0.001**

BCDT, B-cell depleting therapy; DMF, dimethyl fumarate; DMT, therapy naive patients; FG, fingolimod; GA, glatiramer acetate; IFN, interferon-beta; NZ, natalizumab; TFM, teriflunomide; NA, not applicable; *p*-values below 0.05 are shown in bold.

* **Normal range of values:** IgG: 700–1600 mg/dl; IgM: 40–230 mg/dl.

The highest proportion of patients with IgG values below the lower limit of normal was found in patients treated with FG (n = 23; 21.9% of patients), shortly followed by patients treated with NZ (n = 9; 19.6% of patients). Patients treated with BCDT showed the highest proportion of IgM values below the lower limit of normal (according to our laboratory standards), with a total of 32 IgM values below 40mg/dl (26.2% of patients; Table 2).

We performed a multivariate linear regression, comparing Ig levels between therapy groups, in which we included age and gender as covariates. The control group was set as a reference to compare Ig levels between groups.

This resulted in lower IgG and IgM levels in patients treated with FG, NZ, and BCDT than healthy controls (*p* < 0.05) after a median treatment of 37, 30.5, and 23 months respectively (Table 2, Figure 1). Treatment with DMF and TFM lead to relatively low IgG, but not IgM levels (Table 2, Figure 1). The most pronounced effect on IgG was seen in patients treated with FG, with 13.2% lower IgG levels than normal controls, while for IgM the most pronounced effect was seen in patients medicated with NZ (27.5% lower than controls).

DMT-naive patients and patients treated with GA or IFN did not show lower Ig levels than healthy controls (*p* > 0.05, Table 2, Figure 1). Age and male gender were also associated with lower IgG and IgM levels, in accordance with current literature (IgG: *p* = 0.002 and *p* = 0.018; IgM: *p* = 0.014 and *p* < 0.001, respectively).

### Multivariate linear regression of time-dependent Ig reduction within therapy subgroups

**Figure 2.**
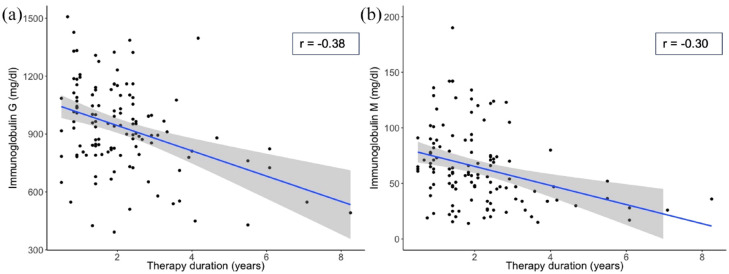
Scatterplots of Ig levels over time under B-cell depleting therapy (BCDT) [a] IgG and [b] IgM. Ig levels (mg/dl) in patients under treatment with BCDT were analyzed by multivariate linear regression in regard to therapy duration and displayed in a scatterplot. The Pearson correlation coefficient r is shown for both IgG and IgM. (a) Analysis of IgG. Estimate: 0.032; *p*-value: < 0.001 (b): Analysis of IgM: Estimate: 0.062; *p*-value: < 0.001.

After stratification of our patients into subgroups based on the medication applied, Ig levels in patients under treatment with FG, NZ, and BCDT were analyzed in regard to therapy duration. Individual multivariate linear regression models were performed, including age, gender, and therapy duration as independent variables and IgG or IgM as the dependent variable. Only BCDT showed a time-dependent decrease of IgG and IgM. Per year IgG and IgM levels decreased by 3.2% and 6.2% respectively (*p* < 0.001; Figure 2). We did not observe major differences between patients treated with rituximab or ocrelizumab with respect to their impact on IgG and IgM levels when the therapy duration was set at 39 months (the highest therapy duration in the ocrelizumab group, Supplementary figure).

### Comparison of IgG subclasses 1–4 between groups

We performed a multivariate linear regression comparing IgG subclasses 1–4 between therapy groups, using the control group as the reference group and including age, gender, and total IgG as further covariates.

The highest levels of IgG1 were found in the control group (median: 794 mg/dl; range: 254–1482 mg/dl), while the lowest levels were in the group of patients under therapy with NZ (median: 526 mg/dl; range: 265–1075 mg/dl). Multivariate linear regression resulted in lower IgG1 levels in patients treated with DMF (*p* < 0.05), with 7.6% lower values than normal controls. Patients treated with BCDT also showed 6% lower IgG1 levels than controls; however, the *p*-value was exactly 0.05, thus closely missing statistical significance.

IgG2 levels were found to be 10.6% lower in patients under therapy with FG (*p* < 0.05) than in controls.

**Figure 3.**
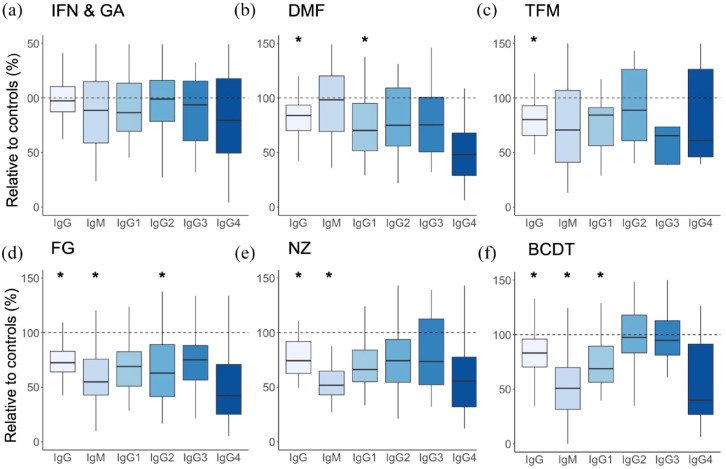
Ig level reduction relative to controls in percent by therapy group Ig levels (IgG, IgM, and IgG subclasses) are demonstrated as variation from controls in percent and divided by therapy group. *Statistical significance compared to controls (see Table 2 and Table 3) BCDT, B-cell depleting therapy; DMF, dimethyl fumarate; FG, fingolimod; GA, glatiramer acetate; IFN, interferon-beta; NZ, natalizumab; TFM, teriflunomide.

IgG3 and IgG 4 levels did not differ between groups (*p* > 0.05). Comparison between controls and DMT-naive MS patients did not result in differences of any IgG subclasses. Age and gender did not seem to be associated with lower IgG subclasses 1–3. Interestingly, female gender was associated with lower IgG4 levels in this subset of patients. For further details, please see Table 3. Furthermore, we visualized IgG subclass levels of each therapy group as percent from controls in Figure 3.

**Table 3.** Median IgG subclass levels 1–4 by therapy group and results of the multivariate linear regression.

		IgG1	IgG2	IgG3	IgG4
Total	Median[mg/dl (range)]	622 (226–1482)	161 (29–549)	43 (10–207)	21 (1–179)
	Estimate	NA	NA	NA	NA
	*p*-value	NA	NA	NA	NA
Controls	Median [mg/dl (range)]	794 (254–1482)	172 (58–394)	48 (14–207)	25 (3–179)
	Estimate	Reference	Reference	Reference	Reference
	*p*-value	Reference	Reference	Reference	Reference
DMT–	Median [mg/dl (range)]	636 (380–1198)	170 (40–431)	38 (16–93)	29 (4–146)
	Estimate	−0.020	0.003	−0.093	0.120
	*p*-value	0.500	0.950	0.084	0.229
IFN	Median [mg/dl (range)]	746 (379–1302)	195 (32–364)	53 (15–105)	30 (1–101)
	Estimate	−0.005	0.025	0.045	0.028
	*p*-value	0.844	0.592	0.353	0.754
GA	Median [mg/dl (range)]	654 (359–1083)	177 (46–343)	48 (18–168)	32 (8–109)
	Estimate	−0.053	0.020	0.006	0.157
	*p*-value	0.073	0.690	0.910	0.106
DMF	Median [mg/dl (range)]	557 (232–1092)	132 (38–284)	37 (15–93)	16 (2–121)
	Estimate	−0.076	−0.049	−0.067	−0.035
	*p*-value	**0.007**	0.317	0.180	0.710
TFM	Median [mg/dl (range)]	670 (229–931)	153 (69–246)	48 (19–86)	26 (10–75)
	Estimate	−0.041	−0.001	0.010	0.139
	*p*-value	0.386	0.998	0.905	0.375
FG	Median [mg/dl (range)]	548 (226–1127)	109 (29–339)	38 (10–83)	12 (1–105)
	Estimate	−0.018	−0.106	−0.048	−0.106
	*p*-value	0.509	**0.024**	0.313	0.238
NZ	Median [mg/dl (range)]	526 (265–1075)	149 (36–431)	48 (15–145)	19 (3–126)
	Estimate	−0.032	0.016	0.005	0.063
	*p*-value	0.276	0.761	0.927	0.520
BCDT	Median [mg/dl (range)]	546 (313–1025)	173 (60–549)	49 (29–123)	15 (2–101)
	Estimate	−0.060	0.048	0.089	−0.089
	*p*-value	**0.050**	0.371	0.135	0.385

BCDT, B-cell depleting therapy; DMF, dimethyl fumarate; DMT, therapy naive patients; FG, fingolimod; GA, glatiramer acetate; IFN, interferon-beta; NA: not applicable; NZ, natalizumab; TFM, teriflunomide; p-values below 0.05 are shown in bold.

## Discussion

In this explorative study, we investigated total IgG and IgM as well as IgG subclasses in patients with MS. Treatment naive MS patients did not differ in their Ig levels from controls, challenging the hypothesis of a previous study that MS in itself may cause hypoimmunoglobulinemia.^13^ We observed lower levels of IgM and IgG under therapy with FG, NZ, and BCDT compared with healthy controls. Treatment with DMF and TFM lead to significantly lower IgG but had no impact on IgM levels. DMF and BCDT also resulted in lower IgG1, while FG resulted in lower levels of IgG2. Interestingly, our results do not show a reduction in IgG4 under BCDT which might be attributed to the low sample size of 26 patients under therapy with BCDT.

Our results confirm the findings of previous studies in regard to the effects of BCDT, NZ, and FG, while DMF and TFM were not known to lead to lower Ig levels compared with controls.^5,7–9,12–14,18^ PROCLAIM, a 96-week phase 3b study even reported stable IgM, IgG, and IgG subclass1–4 levels under DMF treatment.^5,7–9,12–14,18^ This may possibly be due to our longer follow-up time with 34 of our 68 patients (50%) under DMF showing a therapy duration of ⩾96 weeks. Our longest therapy duration within this group was 360 weeks.

With the exception of rituximab and DMF, so far, no data on the effect of DMTs on the individual IgG subclasses have been published. Of all DMTs included in this study, only BCDT showed a time-dependent decrease of IgG and IgM.

Low immunoglobulin levels may result in a higher susceptibility to infectious diseases but since we concentrated on laboratory findings and collected no data on infection rates, conclusions about the correlation between low immunoglobulin levels under different immunotherapies such as BCDT and an increased risk of infection cannot be drawn from this study. However, based on the findings of Perriguey *et al.*^19^ and Barmettler *et al.*^7^ demonstrating that low IgG levels are associated with a higher risk of infection in patients under rituximab, regularly monitoring of Ig levels in patients receiving BCDT can be useful to allow an earlier identification of patients at risk for infectious complication.^7,19^

As stated within the methods section, we decided to collectively analyze the effects of rituximab (*n* = 74) and ocrelizumab (*n* = 48) on the Ig levels of our cohort to increase statistical power. As both DMTs are monoclonal CD20 antibodies, no difference in their effects on Ig levels should be expected. A linear multivariate regression including individual analysis of these two DMTs on IgG and IgM levels supported this hypothesis. Sub-analysis of their effect on IgG subclasses was not performed due to insufficient sample size.

The largest strength of our analysis lies in our large cohort of patients with balanced participant numbers between groups, which increases the power of our findings. However, in regard to IgG subclasses, only a subset of patients with conserved sera underwent ELISA testing, leading to a smaller cohort (Table 1). For some DMTs only, a small number of sera were available for testing, limiting the statistical power of analysis for these groups.

Furthermore, relatively short median therapy duration may hide possible time dependency of Ig reductions so that further studies with longer exposure to medication are warranted to validate these findings. Due to the explorative nature of this study, we did not correct for multiple testing. Naturally, this should be taken into account when performing validation studies.

A further limitation of our study is the absence of immunoglobulin level data before start of each treatment which would be a prerequisite to compare baseline and post-treatment immunoglobulin levels.

As we have already mentioned above, another limitation is the lack of data regarding the rate of infections under different immunotherapies. For future studies, we would, therefore, recommend to also include clinical data to identify the relevance of low immunoglobulin levels in clinical practice.

## Supplemental Material

sj-docx-2-tan-10.1177_17562864231162661 – Supplemental material for The impact of disease-modifying therapies on immunoglobulin blood levels in patients with multiple sclerosis: a retrospective cross-sectional study Click here for additional data file. Supplemental material, sj-docx-2-tan-10.1177_17562864231162661 for The impact of disease-modifying therapies on immunoglobulin blood levels in patients with multiple sclerosis: a retrospective cross-sectional study by Ana Klein, Martina Flaskamp, Achim Berthele, Friederike Held, Harisa Muratovic and Bernhard Hemmer in Therapeutic Advances in Neurological Disorders

sj-jpg-1-tan-10.1177_17562864231162661 – Supplemental material for The impact of disease-modifying therapies on immunoglobulin blood levels in patients with multiple sclerosis: a retrospective cross-sectional study Click here for additional data file. Supplemental material, sj-jpg-1-tan-10.1177_17562864231162661 for The impact of disease-modifying therapies on immunoglobulin blood levels in patients with multiple sclerosis: a retrospective cross-sectional study by Ana Klein, Martina Flaskamp, Achim Berthele, Friederike Held, Harisa Muratovic and Bernhard Hemmer in Therapeutic Advances in Neurological Disorders

## Declarations

**Ethics approval and consent to participate:** This study was approved by the ethics committee of the Technical University of Munich (number 2022-375-S-NP). All participants provided written informed consent.

**Consent for publication:** Not applicable.

**Availability of data and materials:** The data that support the findings of this study are available from the corresponding author upon reasonable request. The data are not publicly available to protect the privacy of the participating patients.
