# Supplementary material for: The impact of disease-modifying therapies on immunoglobulin blood levels in patients with multiple sclerosis: a retrospective cross-sectional study
Source: Ther Adv Neurol Disord. 2023 Apr 17;16:17562864231162661. doi: 10.1177/17562864231162661 (PMC10126592; doi:10.1177/17562864231162661)
Supplement: sj-docx-2-tan-10.1177_17562864231162661 – Supplemental material for The impact of disease-modifying therapies on immunoglobulin blood levels in patients with multiple sclerosis: a retrospective cross-sectional study [file sj-docx-2-tan-10.1177_17562864231162661.docx]

**Supplemental material**

**Median Ig levels by therapy group including individual analysis of Rituximab and Ocrelizumab and results of the multivariate linear regression**

|  |  | **Median**  **[mg/dl (range)]** | **Below lower limit of normal [n (%)]** | **Estimate** | **p-value** |
| --- | --- | --- | --- | --- | --- |
| **Total** | **IgG** | 986 (392-1858) | 58 (9.6) | NA | NA |
|  | **IgM** | 83 (11-432) | 64 (10.6) | NA | NA |
| **Controls** | **IgG** | 1134 (727-1853) | 0 (0.0) | Reference | Reference |
|  | **IgM** | 114 (30-296) | 0 (0.0) | Reference | Reference |
| **DMT-** | **IgG** | 1136 (663-1858) | 1 (1.4) | -0.001 | 0.968 |
|  | **IgM** | 122 (45-302) | 0 (0.0) | 0.024 | 0.560 |
| **IFN** | **IgG** | 1132 (812-1598) | 0 (0.0) | -0.008 | 0.598 |
|  | **IgM** | 94 (31-223) | 4 (6.8) | -0.017 | 0.646 |
| **GA** | **IgG** | 1088 (705-1816) | 0 (0.0) | -0.145 | 0.128 |
|  | **IgM** | 127 (27-373) | 0 (0.0) | 0.187 | 0.402 |
| **DMF** | **IgG** | 956 (480-1785) | 6 (8.8) | -0.068 | **<0.001** |
|  | **IgM** | 116 (41-432) | 0 (0.0) | 0.019 | 0.644 |
| **TFM** | **IgG** | 909 (546-1387) | 3 (12.5) | -0.085 | **<0.001** |
|  | **IgM** | 83 (15-189) | 0 (0.0) | -0.107 | 0.053 |
| **FG** | **IgG** | 821 (480-1451) | 23 (21.9) | -0.133 | **<0.001** |
|  | **IgM** | 64 (11-232) | 16 (15.2) | -0.205 | **<0.001** |
| **NZ** | **IgG** | 846 (561-1816) | 9 (19.6) | -0.104 | **<0.001** |
|  | **IgM** | 59 (11-137) | 7 (15.2) | -0.281 | **<0.001** |
| **RTX** | **IgG** | 825 (392-1397) | 13 (27.1) | -0.138 | **<0.001** |
|  | **IgM** | 47 (27-134) | 20 (41.7) | -0.368 | **<0.001** |
| **OCR** | **IgG** | 1005 (425-1508) | 3 (4.1) | -0.047 | **0.006** |
|  | **IgM** | 69 (14-280) | 12 (16.2) | -0.218 | **<0.001** |

**Supplementary figure: Scatterplots of Ig levels over time under rituximab and ocrelizumab. [A] IgG and [B] IgM]**

Ig levels (mg/dl) in patients under treatment with rituximab (dark blue) and ocrelizumab (light blue) were analyzed by multivariate linear regression in regard to therapy duration and displayed in a scatterplot. The Pearson correlation coefficient r is shown for both IgG and IgM. Therapy duration was adjusted to 39 months, according to the maximum therapy duration within the ocrelizumab group.
