# Supplementary figures and images for: The impact of disease-modifying therapies on immunoglobulin blood levels in patients with multiple sclerosis: a retrospective cross-sectional study
Source: Ther Adv Neurol Disord. 2023 Apr 17;16:17562864231162661. doi: 10.1177/17562864231162661 (PMC10126592; doi:10.1177/17562864231162661)

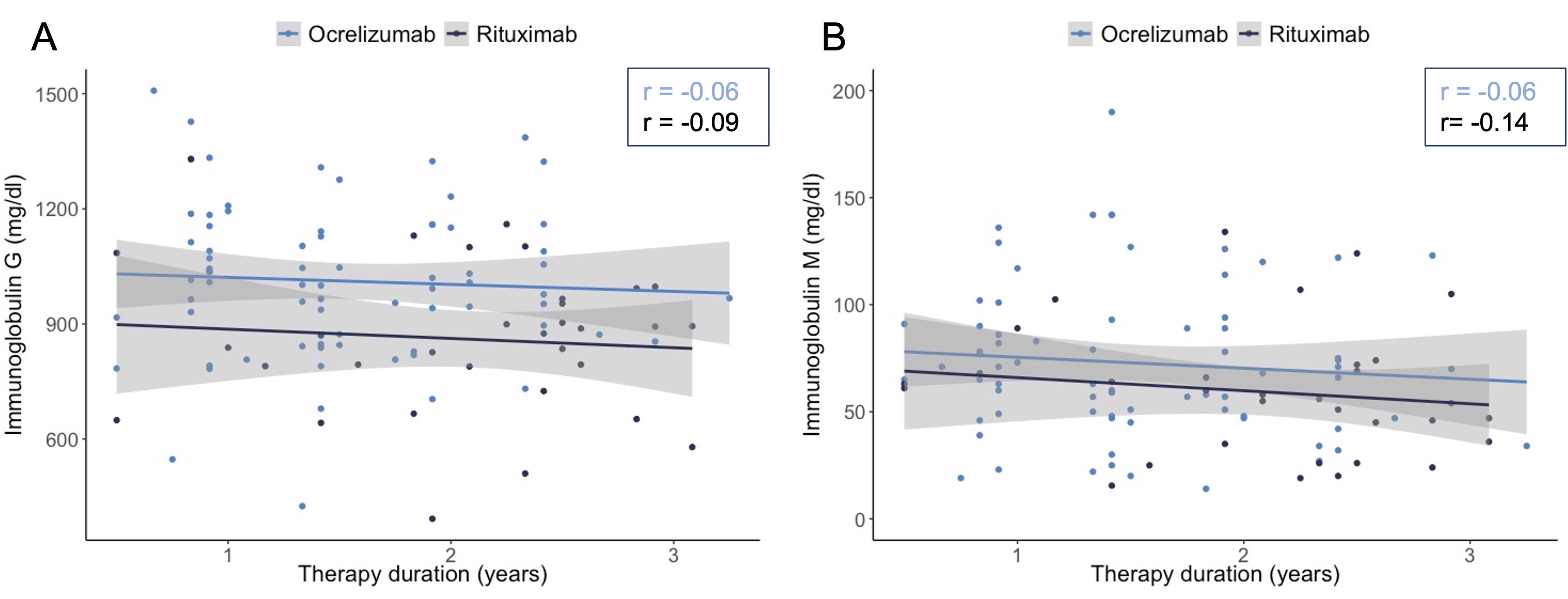

Supplement: sj-jpg-1-tan-10.1177_17562864231162661 – Supplemental material for The impact of disease-modifying therapies on immunoglobulin blood levels in patients with multiple sclerosis: a retrospective cross-sectional study [file sj-jpg-1-tan-10.1177_17562864231162661.jpg]
